# Bat-borne H9N2 influenza virus evades MxA restriction and exhibits efficient replication and transmission in ferrets

**DOI:** 10.1038/s41467-024-47455-6

**Published:** 2024-04-25

**Authors:** Nico Joel Halwe, Lea Hamberger, Julia Sehl-Ewert, Christin Mache, Jacob Schön, Lorenz Ulrich, Sten Calvelage, Mario Tönnies, Jonas Fuchs, Pooja Bandawane, Madhumathi Loganathan, Anass Abbad, Juan Manuel Carreño, Maria C. Bermúdez-González, Viviana Simon, Ahmed Kandeil, Rabeh El-Shesheny, Mohamed A. Ali, Ghazi Kayali, Matthias Budt, Stefan Hippenstiel, Andreas C. Hocke, Florian Krammer, Thorsten Wolff, Martin Schwemmle, Kevin Ciminski, Donata Hoffmann, Martin Beer

**Affiliations:** 1 Institute of Diagnostic Virology, Friedrich-Loeffler-Institut, 17493 Greifswald, Insel Riems Germany; 2 Institute of Virology, Medical Center-University of Freiburg, 79104 Freiburg, Germany; 3 Faculty of Medicine, University of Freiburg, 79104 Freiburg, Germany; 4 Department of Experimental Animal Facilities and Biorisk Management, Friedrich-Loeffler-Institut, 17493 Greifswald, Insel Riems Germany; 5 Unit 17, Influenza and Other Respiratory Viruses, Robert Koch-Institut, Seestraße 10, 13353 Berlin, Germany; 6 HELIOS Clinic Emil von Behring, Department of Pneumology and Department of Thoracic Surgery, Chest Hospital Heckeshorn, Berlin, Germany; 7 Department of Microbiology, Icahn School of Medicine at Mount Sinai, New York, NY 10029 USA; 8 Center for Vaccine Research and Pandemic Preparedness (C-VaRPP), Icahn School of Medicine at Mount Sinai, New York, NY USA; 9 Department of Pathology, Molecular and Cell Based Medicine, Icahn School of Medicine at Mount Sinai, New York, NY USA; 10 Division of Infectious Diseases, Department of Medicine, Icahn School of Medicine at Mount Sinai, New York, NY USA; 11 The Global Health and Emerging Pathogens Institute, Icahn School of Medicine at Mount Sinai, New York, NY USA; 12 Center of Scientific Excellence for Influenza Virus, Institute of Environmental Research and Climate Changes, National Research Centre, Giza, Egypt; 13 Human Link DMCC, Dubai, United Arab Emirates; 14 Department of Infectious Diseases and Respiratory Medicine, Charité—Universitätsmedizin Berlin, corporate member of Freie Universität Berlin and Humboldt Universität zu Berlin, Berlin, Germany

**Keywords:** Virology, Viral infection, Influenza virus, Mouse

## Abstract

Influenza A viruses (IAVs) of subtype H9N2 have reached an endemic stage in poultry farms in the Middle East and Asia. As a result, human infections with avian H9N2 viruses have been increasingly reported. In 2017, an H9N2 virus was isolated for the first time from Egyptian fruit bats (*Rousettus aegyptiacus*). Phylogenetic analyses revealed that bat H9N2 is descended from a common ancestor dating back centuries ago. However, the H9 and N2 sequences appear to be genetically similar to current avian IAVs, suggesting recent reassortment events. These observations raise the question of the zoonotic potential of the mammal-adapted bat H9N2. Here, we investigate the infection and transmission potential of bat H9N2 in vitro and in vivo, the ability to overcome the antiviral activity of the human MxA protein, and the presence of N2-specific cross-reactive antibodies in human sera. We show that bat H9N2 has high replication and transmission potential in ferrets, efficiently infects human lung explant cultures, and is able to evade antiviral inhibition by MxA in transgenic B6 mice. Together with its low antigenic similarity to the N2 of seasonal human strains, bat H9N2 fulfils key criteria for pre-pandemic IAVs.

## Abstract (web summary)

In this study, the authors report that bat H9N2 influenza A virus replicates and transmits in ferrets, efficiently infects human lung explant cultures, evades MxA antiviral activity in mice, and has low antigenic similarity to seasonal N2, meeting pre-pandemic criteria.

## Introduction

Influenza A viruses (IAVs) are highly infectious viral pathogens that can cross interspecies barriers and infect a wide range of avian and mammalian species^1^. Although bats have long been known to be reservoirs for a variety of viruses^2^, they were only recently found to also harbor IAVs^3,4^. While H17N10 and H18N11 strains were first identified in Central and South American bat species^3,4^, the H9N2 virus A/bat/Egypt/381OP/2017, designated as bat H9N2, was recently isolated from Egyptian fruit bats (*Rousettus aegyptiacus*) in the Nile Delta region^5^. Phylogenetic analyses suggest that this Old World bat H9N2 virus is distinct from the New World bat IAVs H17N10 and H18N11, and emerged as a reassortant from an ancestral bat backbone and avian IAV H9 and N2 segments^6^. Avian H9N2 viruses were first isolated from turkeys in North American poultry farms in 1966^7^ and subsequently became endemic in poultry farms in many countries in the Middle East and Asia^8,9^. Since then, avian H9N2 viruses have become widespread and have undergone extensive reassortment with other circulating avian IAVs, resulting in at least 74 different lineages^10^. Over the past two decades, avian H9N2 infections have been recorded in swine populations and mink farms^11,12^. Furthermore, since 1998, the WHO has reported 82 human spill-over infections with avian IAVs in China or Cambodia, resulting in mild to severe disease^13^. Interestingly, sero-epidemiological data from Ghanaian straw-colored fruit bats showed a high prevalence of H9-specific antibodies (30%)^14^, and bat H9N2 was also detected in Egyptian fruit bats from South Africa^15^, suggesting widespread circulation of bat H9N2 in African bat populations. Similar to avian H9N2, bat H9N2 initiates infection by utilizing avian IAV-like α2,3 sialic acid receptors, and replicates in mice, but not in adult chickens^5^. Here, we investigated whether bat H9N2 is of zoonotic concern. We show that the bat H9N2 IAV isolated from Egyptian fruit bats exhibits robust replication and transmission in ferrets, effectively infects human lung cells, evades MxA antiviral activity, and has low antigenic similarity to seasonal human strains, indicating its potential as a pre-pandemic influenza strain.

## Results and discussion

As H9N2 viruses were originally isolated from turkeys^7^, we first determined the replication properties of bat H9N2 in 1-day-old turkeys. Following oro-nasal inoculation, bat H9N2 replicated efficiently to 10^5^–10^7^ copies mL^−1^ at 1 day post infection (dpi; Supplementary Fig. 1a). Thereafter, viral loads rapidly decreased but again reached titers of 5 × 10^5^ copies mL^−1^ between 5 and 8 dpi. Infectious virus was isolated from oral swabs collected at 5 dpi (Supplementary Fig. 1a). At 11 dpi, all but one oral swab was negative for viral RNA (Supplementary Fig. 1a) and no viral RNA was detected in cloacal swabs at any time point measured. All turkey hatchlings seroconverted with antibodies targeting the viral nucleoprotein (NP) at 21 dpi (Supplementary Fig. 1b, c), demonstrating that bat H9N2 maintained its ability to replicate in turkeys. In contrast, and in agreement with previous reports^5^, bat H9N2 failed to replicate efficiently in 1-day-old chicken and did not elicit an antibody response (Supplementary Fig. 1d, e). In the future, the molecular species differences that allow the bat H9N2 to replicate efficiently in turkeys but not chickens need to be investigated in more detail.

**Fig. 1:**
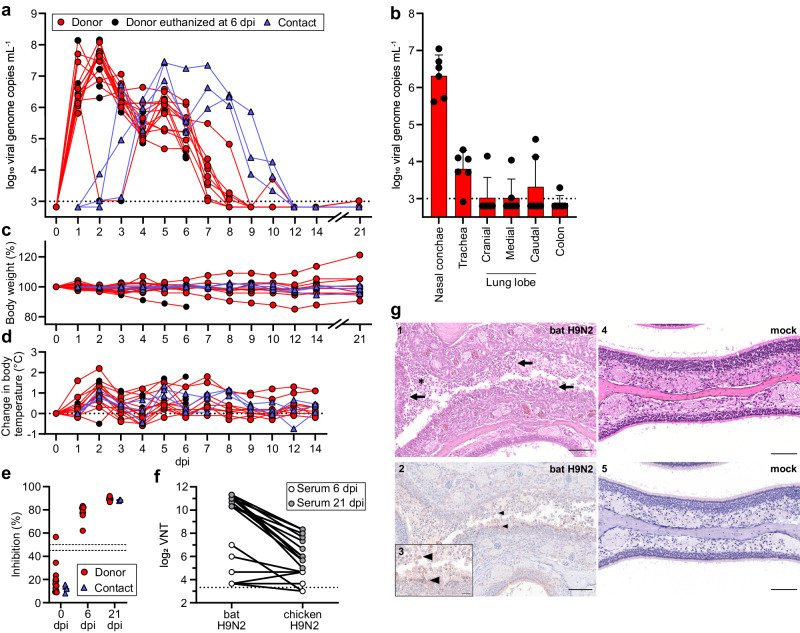
Ferrets are highly susceptible to bat H9N2. **a** Ferrets (*n* = 15) were inoculated with 10^4.8^ TCID_50_ of bat H9N2 IAV per animal. At 1 dpi, direct contact animals (*n* = 3) were co-housed. Donor ferrets euthanized at 6 dpi are indicated. Viral shedding was measured by nasal lavage. Dashed line indicates detection limit. **b** Organs collected from euthanized ferrets (*n* = 6) at 6 dpi with bat H9N2 were tested by RT-qPCR to determine viral genome copies. Dashed line indicates detection limit. Data are mean ± SD. **c** Changes in body weight relative to 0 dpi of bat H9N2-infected (*n* = 15) and contact (*n* = 3) ferrets were monitored throughout the course of the experiment. **d** Changes from the baseline body temperatures of donor and naïve contact ferrets were monitored from 0 to 14 dpi. **e** Ferret serum antibody titers in an IAV NP-specific ELISA at the indicated time points (0 dpi *n* = 18, 6 dpi *n* = 6, 21 dpi *n* = 12). Dashed lines indicates threshold between 45% and 50% inhibition. Mean antibody titers are indicated. Note that one ferret showed weak reactivity in the pre-experimental NP-ELISA, but was included in the study because of its low seropositivity, which could be due to a previously unrecognized IAV infection or an unspecific ELISA reactivity. There was no indication of H9N2 serology prior to inoculation. **f** Ferret neutralizing antibody titers (*n* = 18) against bat H9N2 and chicken H9N2. **g** Histopathologic findings with detection of viral antigen in the nasal mucosa of bat H9N2-infected ferrets (*n* = 6, panels 1–3) at 6 dpi or mock-infected ferret (*n* = 1, panels 4 and 5). Acute severe rhinitis with diffuse necrosis of the olfactory epithelium (arrow) and infiltrating neutrophils (asterisk) (1). Intralesional viral antigen (NP) is abundant in degenerated and desquamated epithelial cells (arrowhead) (2). The inset (3) is a higher magnification of the center of the image (2). No pathology was observed in the mock-infected ferret (4, 5). Representative images are shown. Scale bar, 100 µm (main panels), 25 µm (inset). Source data are provided as a Source Data file.

In order to assess the zoonotic potential and transmissibility of bat H9N2 in the model most relevant to humans, we infected 15 donor ferrets and co-housed three naïve contact animals from 1 to 12 dpi (Fig. 1a and Supplementary Fig. 1f). Quantification of viral RNA obtained from nasal lavages revealed substantial viral replication (10^6^–10^8^ copies mL^−1^) within the first 2 days after infection and continuous shedding of viral genomes and infectious virus up to 8 dpi (Fig. 1a and Supplementary Fig. 1g). Strikingly, all contact animals acquired a viral infection from donor ferrets after co-housing with peak titers (10^7^ copies mL^−1^) at 4 days post exposure (dpe) (Fig. 1a). To determine whether bat H9N2 replication is limited to the upper respiratory tract, we next measured viral titers in the organs of six donor ferrets euthanized at 6 dpi (Fig. 1b). While all ferrets had substantial viral genome copies in the nasal conchae and five of six animals had moderate levels in the trachea, one of six ferrets had moderate viral genome levels in the cranial lung lobe, two in the medial and caudal lung lobes, and one ferret even had low viral copies in the colon (Fig. 1b). We did not observe severe body weight loss in most donor and any contact ferrets, although two donor animals exhibited ~15% weight loss at 6 and 12 dpi (Fig. 1c), which was most likely unrelated to infection. Elevated body temperatures were observed at 2 dpi in 14 of 15 donor ferrets (Fig. 1d) and all contact animals had elevated body temperatures at 1 dpe (Fig. 1d). Seroconversion with NP-specific antibodies was detected as early as 6 dpi in donor ferrets, and all donor and contact ferrets examined at 21 dpi exhibited a robust NP-specific antibody response (Fig. 1e). Furthermore, at 21 dpi we determined antibodies with a strong neutralizing capacity against bat H9N2 and some degree of cross-neutralization against the avian H9N2 A/layer chicken/Bangladesh/VP02-plaque/2016 isolate (Fig. 1f). Histopathological examination revealed severe purulent to necrotizing rhinitis with viral antigen in the epithelia of the respiratory and olfactory tract in all infected ferrets at 6 dpi (Fig. 1g). We observed mild infection-induced changes characterized by focal to oligofocal epithelial necrosis and mild infiltration of the lamina propria in the trachea of four animals. In accordance with the low viral genome copies detected in the ferret lungs, no influenza-associated lung pathology was detected, which is in contrast to lung pathology of ferrets after infection with e.g. reassorted H9N2- or highly pathogenic avian influenza strains^16–19^.

**Fig. 2:**
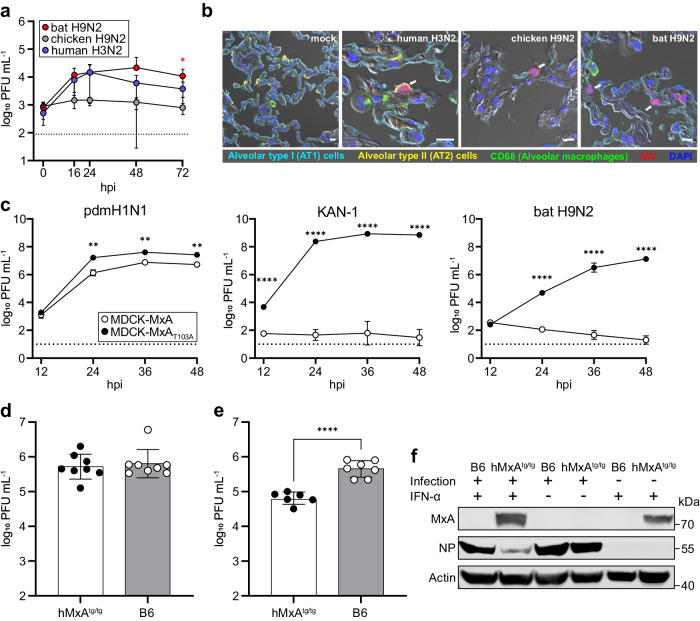
Bat H9N2 replicates in human lung explants and suppresses induction of MxA in MxA-transgenic mice. **a** Human lung tissue explants (*n* = 4) were infected with human H3N2, chicken H9N2 or bat H9N2 with 1 × 10^6^  PFU, and viral titers were determined at the indicated time points. Error bars indicate standard deviation and statistical analysis was performed using non-paired, non-parametric Kruskal-Wallis test (*p = 0.0324). Data are mean ± SD of *n* = 4 independent experiments. Dashed line indicates detection limit (**b**) At 24 hpi, human lung explants were stained for alveolar type I (AT1) (cyan) and type II (AT2) cells (yellow), CD68 indicating alveolar macrophages (green) and IAV antigens (red). Note, in chicken H9N2 and bat H9N2 infected cells, AT2 labeling was omitted for better visualization. White arrows indicate infected cells. Scale bar, 10 µm. **c** MDCK cells overexpressing MxA or inactive MxA_T103A_ were infected with human-adapted pdmH1N1, avian KAN-1 (H5N1) or bat H9N2 at an MOI of 0.001, and viral titers were determined at the indicated time points. Data are mean ± SD of *n* = 3 independent experiments; statistical analysis was performed using two-tailed *t*-tests; **P = 0.01; ****P = 0.0001. Dashed line indicates detection limit. **d** hMxA^tg/tg^ (*n* = 8) or wild-type B6 mice (*n* = 8) were infected with 1 × 10^4^  PFU. Lung viral titers were determined 3 dpi. **e** hMxA^tg/tg^ (*n* = 6) or wild-type B6 mice (*n* = 7) were pretreated with IFN-α 18 h prior to infection with 1 × 10^4^ PFU. Lung viral titers were determined 3 dpi. Data are mean ± SD; statistical analysis was performed using two-tailed *t*-tests; ****P = 0.0001. **f** MxA, NP and actin protein levels in homogenized lungs from IFN-α pretreated or infected mice from (**d**,**e**) were detected by Western blot. Source data are provided as a Source Data file.

Because severe courses of influenza in humans almost always affect the lower respiratory tract^20^, we next infected human ex vivo lung cultures with bat H9N2, a prototypic human seasonal H3N2 isolate (A/Panama/2007/1999) or chicken H9N2 and determined viral growth properties. Intriguingly, bat H9N2 replicated to comparable or even higher viral titers than human H3N2, reaching peak titers of 3 × 10^4^ plaque-forming units (PFU) mL^−1^ at 48 h post infection (hpi; Fig. 2a). In contrast, chicken H9N2 showed minimal viral replication in human lung tissue. Immunostaining of lung explants at 24 hpi revealed that all viruses infected alveolar type II cells (Fig. 2b), which is the primary cellular tropism of IAV in the lung^21^.

Next, we studied whether bat H9N2 is able to escape human MxA, a crucial innate antiviral factor which restricts IAVs by inhibiting their polymerase activity^22^. Human-adapted IAVs, such as the pandemic H1N1 virus A/Hamburg/4/2009 (pdmH1N1), acquire characteristic clusters of adaptive mutations in NP that enable escape from MxA^22,23^, whereas such clusters are virtually absent in IAVs of avian origin including the highly pathogenic H5N1 strain A/Thailand/1(KAN-1)/2004 (KAN-1). Bat H9N2 NP also lacks the residues described as conferring MxA resistance (Supplementary Fig. 2a). Thus, as expected, bat H9N2 exhibited a high degree of MxA-sensitivity as demonstrated by infecting MDCK cells stably overexpressing either wild-type MxA (MDCK-MxA) or the antivirally inactive MxA_T103A_ variant (MDCK-MxA_T103A_)^24^. While pdmH1N1, KAN-1 and bat H9N2 replicated efficiently in MDCK-MxA_T103A_ cells to titers between 1.3 × 10^7^ and 7 × 10^8^ PFU mL^-1^ at 48 hpi (Fig. 2c), KAN-1 was nearly completely inhibited in MDCK-MxA cells whereas peak titers of pdmH1N1 decreased only 5-fold. Replication of bat H9N2 was potently restricted in the presence of MxA as illustrated by residual viral titers ≤ 10^2^  PFU mL^−1^ between 24–48 hpi (Fig. 2c).

To assess the importance of MxA in controlling bat H9N2 in vivo, we intranasally infected wild type C57BL/6 (B6), which lack a functional Mx protein, and human MxA-transgenic (hMxA^tg/tg^) mice with bat H9N2. Surprisingly, lung viral titers were similar in both B6 and MxA-transgenic mice^25^ at 3 dpi (5 × 10^5^ PFU mL^-1^; Fig. 2d), as confirmed by comparable NP levels in lung homogenates detected by Western blotting (Fig. 2f). Interestingly, MxA expression was not observed in the lungs of infected hMxA^tg/tg^ mice, but could be potently induced by IFN-α pretreatment 18 h prior to challenge infection with bat H9N2. Under these conditions, we observed induction of MxA (Fig. 2f) and 10-fold lower lung viral lung titers in hMxA^tg/tg^ compared to B6 mice (Fig. 2e), suggesting that MxA, when induced, reduces bat H9N2 replication.

Finally, because there is little serological evidence for H9-specific antibodies in the human population^26,27^, we wondered whether the antibody responses to circulating seasonal H1N1 and H3N2 strains as well as vaccination would be cross-reactive for bat N2. Serum collected from 15 healthy adults before and after seasonal influenza vaccination in 2022/23 revealed no reactivity to bat N2 (Supplementary Fig. 2b,e), but robust reactivity to N2 from the seasonal A/Kansas/14/2017 (H3N2) isolate (Supplementary Fig. 2d).

Our study shows that the Old World bat H9N2 virus meets key characteristics of a pre-pandemic IAV, including replication in and efficient transmission between ferrets, the ability to replicate efficiently in human lung explants and evasion from MxA-mediated restriction. Intriguingly, bat H9N2 exhibits an immediate (at 1 dpe) and highly efficient transmission potential (100%) not previously observed in any avian-derived H9N2 isolate with an α2,3 sialic acid receptor specificty^28–31^. As the bat H9N2 HA specifically binds to avian IAV-like α2,3 sialic acid receptors for cell entry and fails to recognize mammalian-like α2,6 sialic acid receptors^5^, we suggest that infection of ferrets and mice occurs by binding to α2,3 sialic acid receptors, which are also present in low numbers in the upper respiratory tract^32,33^. Thus, the ability of bat H9N2 to replicate in and transmit among ferrets may also allow for spread among and further adaptation to humans. Our data also suggests that bat H9N2 can suppress the expression of MxA, thereby overcoming this important restriction factor for zoonotic spill-over^34^. This is in strong contrast to zoonotic H5N1 and H7N9 viruses of avian origin, which also lack MxA resistance in NP but induce MxA in hMxA^tg/tg^ mice and thus are potently inhibited^25^. Given the ability of bat H9N2 to infect turkey hatchlings, introduction of bat H9N2 into poultry farms and reassortment with avian IAV cannot be ruled out, necessitating increased attention and close monitoring of possible human spill-over infections in Africa.

A further prerequisite of pre-pandemic viruses is their antigenic novelty to the human immune system. Since the human population is presently exposed only to the currently-circulating H1N1 and H3N2 subtypes, a lack of humoral immunity to bat H9N2 is very likely. Indeed, our serological data demonstrates that seasonal influenza vaccines containing H1N1 and H3N2 do not elicit cross-reactive antibodies to the bat N2 protein, substantiating the general pre-pandemic features of bat H9N2.

## Methods

### Virus

The bat-derived H9N2 A/bat/Egypt/381OP/2017 isolate (GenBank accession numbers: MH376902 to MH376909), was propagated in embryonated SPF-chicken eggs for 5 days at 37 °C. Subsequently, the allantoic fluid was harvested and used as virus stock. The chicken H9N2 isolate A/layer chicken/Bangladesh/VP02-plaque/2016 was obtained from the Friedrich-Loeffler-Institut (FLI) virus repository^35^. Virus stocks of the human seasonal A/Panama/2007/1999 (H3N2) isolate were generated by propagation on MDCKII cells. Recombinant A/Hamburg/4/2009 (pdmH1N1) and A/Thailand/1(KAN-1)/2004 (H5N1) were generated utilizing the eight-plasmid pHW2000-based rescue system^36^. All recombinant viruses were plaque purified and then used for stock generation. Stock titers were determined by a plaque assay on MDCKII cells.

### Cells

Madin-Darby Canine Kidney (MDCK) type II cells (Collection of Cell Lines in Veterinary Medicine CCLV RIE1061) were used. Cells were incubated at 37 °C under 5% CO_2_ atmosphere using a mixture of equal volumes of Eagle Minimum Essential Medium (MEM) (Hank’s balanced salts solution) and Eagle MEM (Earle’s balanced salts solution), 2 mM L-Gln, nonessential amino acids, adjusted to 850 mg L^−1^ NaHCO3, 120 mg L^-1^ sodium pyruvate, pH 7.2 with 10% FCS (Bio & Sell GmbH) or without FCS in the presence of tosylsulfonyl phenylalanyl chloromethyl ketone (TPCK)-treated trypsin (Sigma) after virus addition. MDCK-MxA and MDCK-MxA_T103A_ cells were obtained by Jesse D. Bloom (Fred Hutchinson Cancer Research Center, United States)^24^ and were cultured in Dulbecco’s modified Eagle’s medium (DMEM, Gibco, Thermo Fisher Scientific) containing 10% fetal calf serum (FCS), 100 U penicillin and 100 µg streptomycin mL^−1^ at 37 °C and 5% CO_2_.

### Virus infections

MDCK-MxA and MDCK-MxA_T103A_ cells were seeded and grown in 6-well plates. Prior to infection cells were washed with phosphate buffered saline (PBS) containing 0.2% bovine serum albumin (BSA) and then infected with the indicated virus at an MOI of 0.001 in infection medium (DMEM, containing 0.2% BSA and 100 U penicillin and 100 µg streptomycin µL^-1^). For bat H9N2 and pdmH1N1 1 µg mL^-1^ TPCK-treated trypsin was added into the infection medium. Viral titers were determined by plaque assay.

### Infection of human lung explants

Fresh lung explants were obtained from patients suffering from lung carcinoma and undergoing lung resection at local thoracic surgeries. Written informed consent was obtained from all patients and the study was approved by the ethics committee at the Charité clinic (project EA2/079/13). Tumor-free peripheral lung tissue was cut into small pieces and incubated overnight at 37 °C with 5% CO_2_ in Roswell Park Memorial Institute (RPMI) 1640 medium. The next day, lung tissue was infected with 1 × 10^6^ PFU of either human seasonal H3N2, chicken H9N2 or bat H9N2 for 1.5 h under shaking conditions and excess virus was removed by three washing steps with PBS. Infected lung tissues were incubated at 37 °C and 5% CO_2_ for up to 72 h in RPMI 1640 medium supplemented with 2 mM L-glutamine and 0.3% BA. Viral titers were determined by plaque assay.

### Western blot

Mouse lung samples were incubated at 95 °C in Laemmli buffer and subsequently separated by sodium dodecyl sulfate polyacrylamide gel electrophoresis (SDS–PAGE). Separated protein samples were blotted onto a nitrocellulose membrane. Proteins were detected using specific antibodies against the highly conserved G domain in MxA (M143)23, NP (Gene Tex, GTX125989, 1:1,000), or actin (Sigma-Aldrich, A3853; 1:1,000), respectively. Primary antibodies were detected using peroxidase-conjugated secondary antibodies (Jackson ImmunoResearch, 1:5,000).

### Animal experiment ethics declarations

All ferret and hatchling experiments were evaluated by the responsible ethics committee of the State Office of Agriculture, Food Safety, and Fishery in Mecklenburg–Western Pomerania (LALLF M-V) and gained governmental approval under the registration numbers LVL MV TSD/7221.3-1-029/22 and 7221.3-1-003/22. All mouse experiments were performed in accordance with the guidelines of the German animal protection law and were approved by the state of Baden-Württemberg (Regierungspräsidium Freiburg; reference number: 35-9185.81/G-19/05).

### Animals

One-day-old chickens, 1-day-old turkeys, ferrets as well as C57BL/6 (B6) mice and human MxA transgenic (hMxA^tg/tg^) mice were used for this study. Chicks were bread at the FLI from SPF-chicken eggs (VALOBioMedia, Germany) and 1-day-old turkeys were ordered and shipped on hatching day from a local breeding facility (Bösel) to the FLI. The ferrets were obtained from the in-house breeding program at the FLI. B6 mice were obtained from Janvier and hMxA^tg/tg^ mice were bred in-house at the Institute of Virology, Freiburg. All mice were housed in individually ventilated cages (Green Line, Tecniplast) with stable bedding (2HK: TAPVEI® BEDDING), at a temperature of 20.2 ± 1.1 °C, a humidity of 55% ± 7.1% and a 12 h:12 h light:dark cycle. The mice were fed ad libitum with food made from natural ingredients (Germany, altromin 1314). All animal work was performed in BSL3 containment facilities.

### One-day-old chicken and turkey studies

At the day of hatching, 1-day-old turkeys were inoculated with 10^5^ TCID_50_ per animal and 1-day-old chicks were inoculated with 10^3,9^ TCID_50_ per animal, calculated by back-titration of the original inoculum. All hatchlings were sampled daily via cloacal and oro-pharyngeal swabs until 21 dpi or until the animal samples tested negative in a bat H9N2-specific RT-qPCR. Oro-pharyngeal and cloacal swabs were taken using plain swab sterile paper applicator cotton tips 164 C (Copan, Brescia, Italy). The swabs were immediately transferred into 1 mL of cell culture medium containing 1% Baytril (Bayer, Leverkusen, Germany), 0.5% lincomycin (WDT, Garbsen, Germany) and 0.2% amphotericin/gentamycin (Fisher Scientific Waltham, MA, USA). After euthanasia, nasal conchae and colon organ samples were taken for investigation of viral genome loads via RT-qPCR analysis in the respective organs. Clinical status of the animals was checked daily.

### Ferret study

18 adult ferrets (*Mustela putorius furo*, 10 females and 8 males, aged > 0,5 years) were housed in multi-connected cage units. Before inoculation, blood samples and nasal washings were collected to confirm naivety to IAV of all animals via serological analysis (ELISA) and RT-qPCR. Only one ferret showed some weak reactivity in the pre-experimental NP-ELISA, but was included in the study because of its low seropositivity, which could be due to a previously unrecognized IAV infection or an unspecific ELISA reactivity. However, there was no indication of any H9N2 serology before inoculation. Body weight, body temperature as well as physical condition of all animals was monitored regularly throughout the animal trial. Nasal washing samples were taken under a short-term isoflurane inhalation anesthesia by applying 750 µl of PBS into each nostril and collecting the efflux. Rectal swabs were taken using plain swab sterile paper applicator cotton tips 164 C (Copan). The swabs were immediately transferred into 1 mL of cell culture medium containing 1% Baytril (Bayer, Leverkusen, Germany), 0.5% lincomycin (WDT, Garbsen, Germany) and 0.2% amphotericin/gentamycin (Fisher Scientific Waltham, MA, USA). After 1 week of acclimatization to their new environment (0 dpi), 15 ferrets were intranasally inoculated with 10^4.8^ TCID_50_ per animal in a 200 µL volume (calculated by back-titration of the original material). The inoculum was evenly distributed into each nostril (~100 µl per nostril). At 1 dpi, three naïve contact animals were co-housed in the multi-connected cage units of the donor ferrets to determine direct virus transmission. All animals were sampled via nasal washings and rectal swabs daily until 10 dpi and afterwards every second day until 21 dpi or until the samples tested negative via bat H9N2 specific RT-qPCR analysis. Clinical signs of disease (nasal discharge, reduced activity, fever, neurological symptoms and dyspnea), rectal body temperature and body weight were monitored daily. At 6 dpi, in the acute phase of the infection, six donor ferrets were euthanized and subject to necropsy for pathomorphological investigation and analysis of viral genome loads in the upper and lower respiratory organs, as well as in the intestinal tract. The residual animals were kept until the end of the study at 21 dpi to allow for seroconversion. Nasal conchae organ samples from animals euthanized at 21 dpi were analyzed with a bat H9N2-specific RT-qPCR.

### Mouse study

For infection experiments, 15 B6 mice (7 females and 8 males, aged 6–10 weeks) and 14 hMxA^tg/tg^ mice (7 females and 7 males, aged 6–10 weeks) were anaesthetized with a mixture of ketamine (100 mg per g body weight) and xylazine (5 mg per g body weight) administered intraperitoneally and were subsequently inoculated intranasally with 40 µL of the indicated virus dose diluted in Opti-MEM containing 0.3% BSA. For interferon pretreatment 2 μg per 100 μL IFN-α was administered subcutaneously 18 h prior to challenge with the indicated virus. Throughout the experiment, mice were monitored daily for changes in body weight and other signs of disease. At 3 dpi mice were sacrificed and the lung was dissected. Organs were homogenized in 1 mL PBS by three subsequent rounds of mechanical treatment for 25 s each at 6.5 ms^−1^. Tissue debris was removed by centrifuging homogenates for 5 min at 2.400 × *g* at 4 °C and samples were stored at -80 °C until further processing. Viral organ titers were determined by plaque assay.

### Propagation of bat H9N2 virus isolates from turkey samples

For isolation of bat H9N2 from turkey hatchlings, swab material was used for inoculation of embryonated chicken eggs. Briefly, 200 µl of selected animal samples were transferred into the allantoic cavity of embryonated SPF-chicken eggs (three eggs per sample), followed by incubation for 5 days at 37 °C. Viral genome material was extracted from the allantoic fluid and detected by RT-qPCR analysis.

### Pathomorphology and immunohistochemistry

For the ferret histopathology, nasal conchae, trachea, right cranial, medial and caudal lung lobes as well as the colon were sampled. Tissues were fixed in 10% neutral buffered formalin, embedded in paraffin wax and cut at 3 µm sections. To assess tissue architecture and cell morphology sections were stained with hematoxylin and eosin following standard procedures. For viral antigen detection immunohistochemistry was performed using an in house derived rabbit polyclonal primary antibody directed against the influenza nucleoprotein (NP, 1:750)^37^. Lesions and cellular vial antigen localization were determined and evaluated by a board-certified pathologist (DiplACVP).

To analyze the cellular tropism of IAV infection in human lung tissue samples, tissues were fixed with 4 % paraformaldehyde for 48 h, embedded in paraffin and processed for immunohistochemistry. Lung tissue was then blocked with 5% adequate serum and incubated with primary antibodies directed to CD68 (abcam, Cambridge, UK, 1:50), HT2-280 (terrace biotech, 1:200) and EMP2 (atlas antibodies, 1:50). Viral antigens were stained with polyclonal antibodies to IAV (Serotec, Puchheim, Germany, 1:50) conjugated to a fluorophore (DyLight 488, Thermo Fisher). Primary antibodies were detected using a corresponding secondary labeling kits (OPAL Polaris, Akoyabio) and nuclear counterstaining was performed using DAPI (Sigma, Hamburg, Germany). Finally tissue sections were mounted in Mowiol, and analyzed using a LSM 780 spectral confocal microscope (objectives 63x Plan-Apochromat/oil, NA 1.4, Zeiss, Germany).

### Experimental sample work-up and analysis

Animal organ samples of about 0.1 cm^3^ size were first homogenized in a 2 mL Eppendorf-tube containing 1 mL of Hank’s balanced salts MEM and Earle’s balanced salts MEM (2 mM L-glutamine, 850 mg L^−1^ NaHCO3, 120 mg L^−1^ sodium pyruvate, and 1% penicillin–streptomycin) at 300 Hz using a Tissuelyser II (Qiagen, Hilden, Germany). From each homogenized organ, swab or nasal wash sample, 100 µl was extracted via the NucleoMag Vet kit (Macherey&Nagel, Düren, Germany) according to the manufacturer’s instructions on a Biosprint 96 platform (Qiagen). Viral RNA was detected by RT-qPCR using bat H9N2-specific primers and probes^38^. Absolute quantification was done using a standard of known concentrations, corresponding to the RNA of the original virus used for inoculation. Quantification was established by the QX200 Droplet Digital PCR System in combination with the 1-Step RT-ddPCR Advanced Kit for Probes (BioRad, Hercules, CA, US). Viral titers of ferret nasal washing samples were determined by TCID_50_ endpoint dilution assay on MDCKII cells.

### Human sera collected before and after seasonal influenza vaccination

The observational study protocol IRB−16-00772 was reviewed and approved by the Mount Sinai Hospital Institutional Review Board. All study participants provided written informed consent before biospecimens, and data were collected. Permissions to store and share biospecimen were also obtained from all participants. All specimens were coded before processing and analysis. Whole blood was collected through venipuncture into serum separator tubes and sera were stored at −80 °C until analysis.

### Serology

Serological analysis of blood samples from all animals at respective blood collection time points was performed by using a commercial IAV-specific enzyme-linked immunosorbent assay (ELISA) detecting NP-specific antibodies (ID-Vet, Montpellier, France) according to the manufacturer’s instructions. The antibody titers were expressed as “% inhibition”, which was calculated as ((OD_450_ negative control – OD_450_ sample) / OD_450_ negative control) × 100.

Neutralizing antibody titers were determined in a virus neutralization test (VNT). Briefly, MDCK cells seeded and grown in 96-well plates 24 h before infection. Serum samples were serially diluted in DMEM containing 1 µg mL^−1^ TPCK-treated trypsin and then mixed with 100 TCID_50_ mL^-1^ of either bat or chicken H9N2. After incubation for 2 h at 37 °C and 5% CO_2_, the serum-virus mixture was transferred onto MDCK cells and incubated for 72 h. Neutralization was evaluated by light microscopy for the absence of specific cytopathic effect (CPE), and the corresponding VNT titer was determined from the last serum dilution in which no CPE was observed.

ELISAs with human sera against a recombinant version of the N2 NA of H9N2 virus A/bat/Egypt/381OP/2017 were performed as described in detail before^39^. Recombinant NA from human seasonal H3N2 strain A/Kansas/14/2017 was used to show positive reactivity, recombinant NA from the Wuhan spiny eel influenza virus^40^ (to which humans are naïve) was used as contrast to show negative reactivity. Recombinant proteins were expressed as described previously^41^. Sera collected from 15 study participants before and after receiving the 2022/23 seasonal influenza vaccination were used to determine reactivity to N2 from H9N2, N2 from seasonal H3N2 or to the Wuhan spiny eel influenza virus NA. Monoclonal antibody 1G01^42^ was used as positive control in all cases.

### Reporting summary

Further information on research design is available in the Nature Portfolio Reporting Summary linked to this article.

### Supplementary information

Supplementary Information

Peer Review File

Reporting Summary

### Source data

Source Data

## Data Availability

The data generated in this study are provided in the Supplementary Information/Source Data file. PDB code: 2Q06 and GenBank accession numbers: MH376902 to MH376909 were used in this study. Source data are provided with this paper.
