# Supplementary material for: Bat-borne H9N2 influenza virus evades MxA restriction and exhibits efficient replication and transmission in ferrets
Source: Nat Commun. 2024 Apr 25;15:3450. doi: 10.1038/s41467-024-47455-6 (PMC11045726; doi:10.1038/s41467-024-47455-6)
Supplement: Supplementary file 1 — Supplementary Information [file 41467_2024_47455_MOESM1_ESM.pdf]

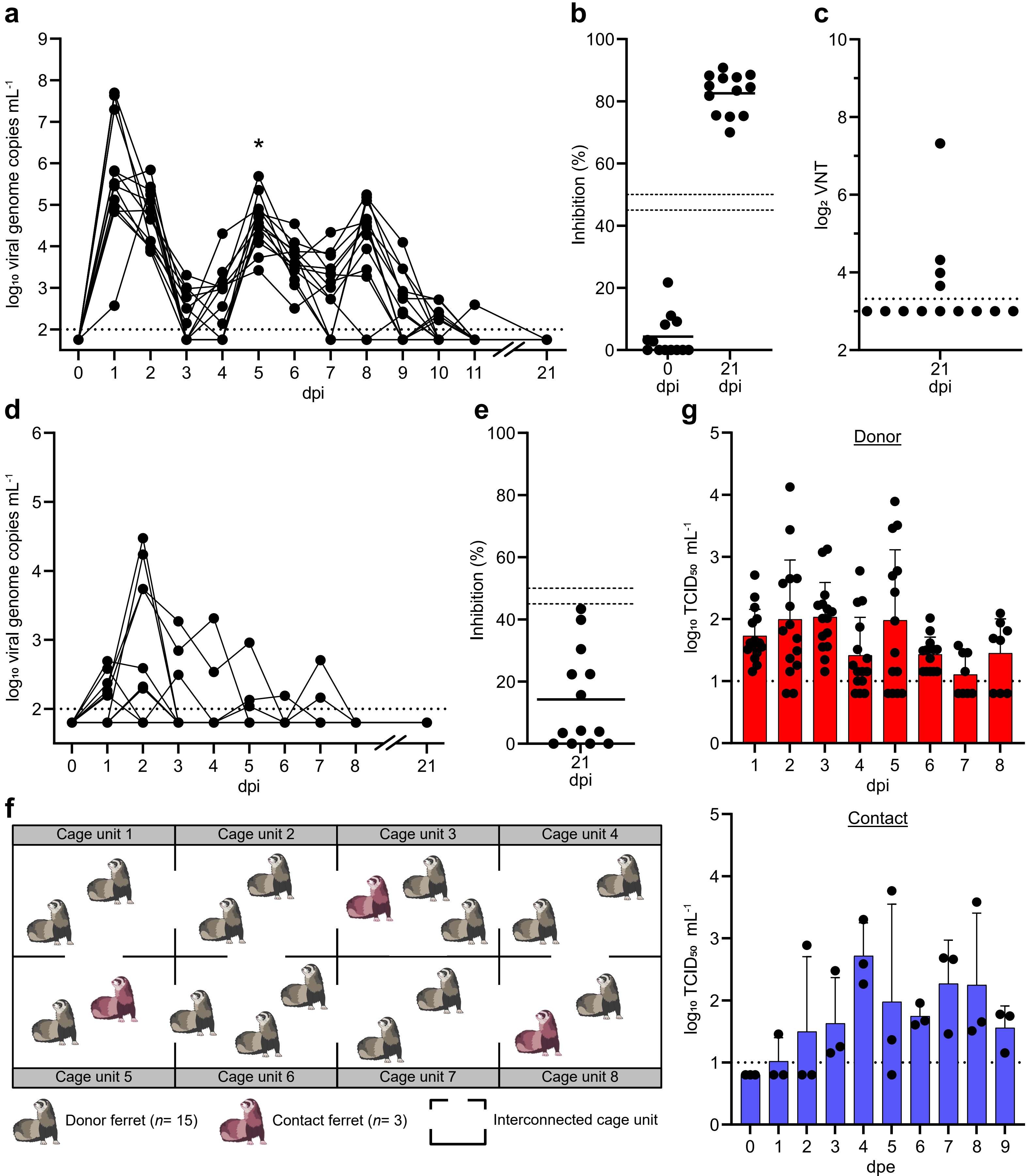
a
log₁₀ viral genome copies mL⁻¹
dpi
*
b
Inhibition (%)
dpi
c
log₂ VNT
dpi
d
log₁₀ viral genome copies mL⁻¹
dpi
e
Inhibition (%)
dpi
g
Donor
log₁₀ TCID₅₀ mL⁻¹
dpi
Contact
log₁₀ TCID₅₀ mL⁻¹
dpe
f
Cage unit 1
Cage unit 2
Cage unit 3
Cage unit 4
Cage unit 5
Cage unit 6
Cage unit 7
Cage unit 8
Donor ferret (n= 15)
Contact ferret (n= 3)
Interconnected cage unit

Supplementary Fig. 1: Bat H9N2 replicates efficiently in turkey but not chicken hatchlings. a Turkey hatchlings (n= 13) were oro-nasally inoculated with bat H9N2 and viral shedding was monitored by RT-qPCR analysis of oro-pharyngeal swab samples. Asterisk indicates successful virus isolation from oral swab material from one turkey tested at 5 dpi. Dashed line indicates detection limit. **b** Serum antibody titers of turkey hatchlings before and after bat H9N2 infection were determined by an IAV NP-specific ELISA. Dashed lines indicates threshold between 45% and 50% inhibition. Mean antibody titers are indicated. **c** Neutralizing antibody titers against bat H9N2. **d** A group of chicken hatchlings (n= 13) was oro-nasally inoculated with bat H9N2 and viral shedding was monitored by RT-qPCR analysis of oro-pharyngeal swab samples. Dashed line indicates detection limit. **e** Serum antibody titers of chicken hatchlings at 21 dpi with bat H9N2 were determined by an IAV NP-specific ELISA. Dashed lines indicate threshold area between 45% and 50% inhibition. Mean antibody titers are indicated. **f** 15 ferrets were directly inoculated with bat H9N2. At 1 dpi, three naïve contact ferrets were housed together with all donor animals in interconnected cage units to allow direct exposure of all ferrets and to study bat H9N2 transmission. **g** Nasal swabs containing shed infectious bat H9N2 virus taken from bat H9N2-infected (n= 15) and contact (n= 3) ferrets at the indicated time points were titrated by endpoint titration on MDCK cells. Dashed line indicates the detection limit. Data are mean ± SD.

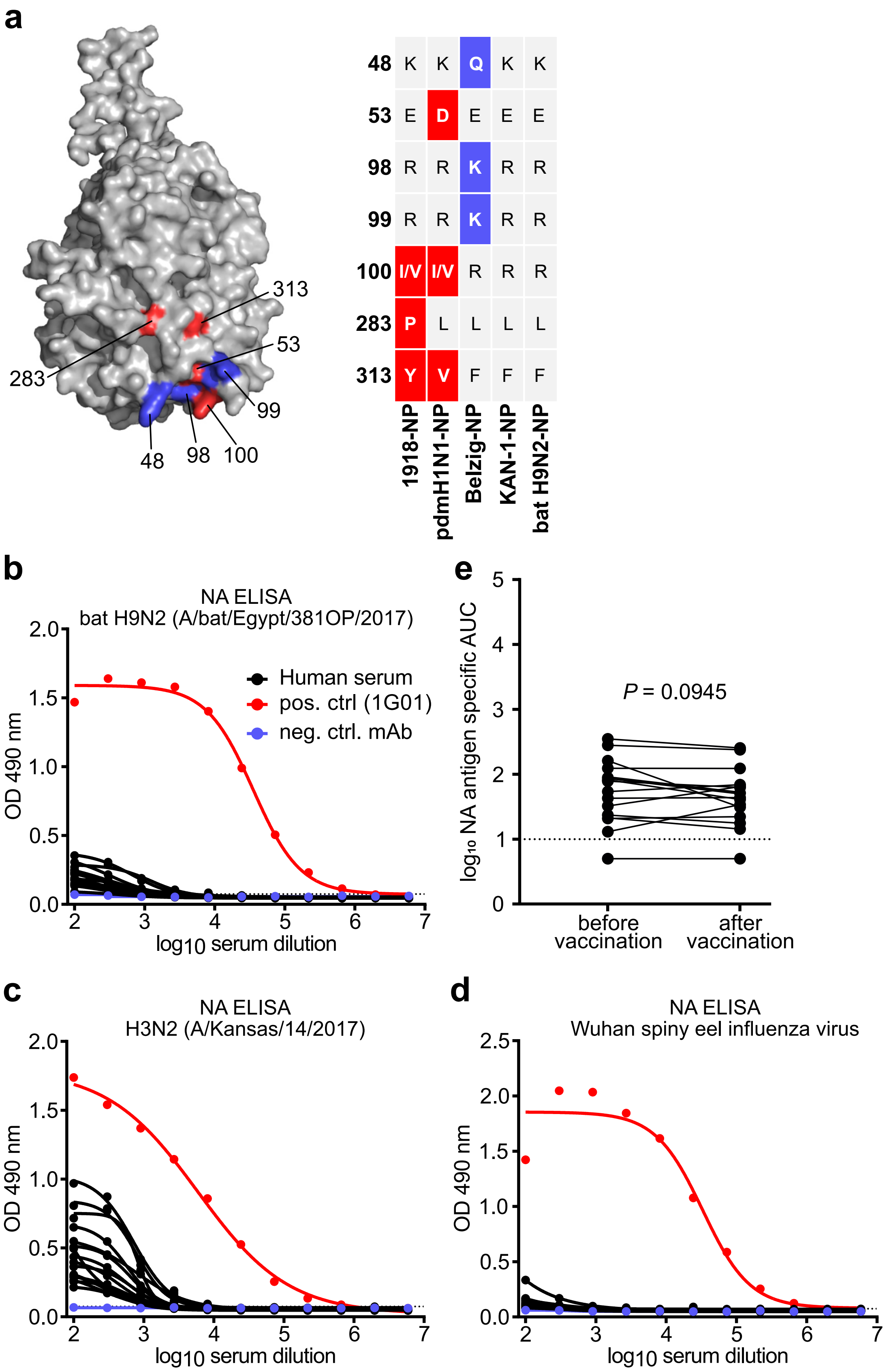

a
48 K K Q K K
53 E D E E E
98 R R K R R
99 R R K R R
100 I/V I/V R R R
283 P L L L L
313 Y V F F F
1918-NP
pdmH1N1-NP
Belzig-NP
KAN-1-NP
bat H9N2-NP
313
283
53
99
48
98
100
b
NA ELISA
bat H9N2 (A/bat/Egypt/381OP/2017)
Human serum
pos. ctrl (1G01)
neg. ctrl. mAb
OD 490 nm
log10 serum dilution
e
P = 0.0945
log₁₀ NA antigen specific AUC
before vaccination
after vaccination
c
NA ELISA
H3N2 (A/Kansas/14/2017)
OD 490 nm
log10 serum dilution
d
NA ELISA
Wuhan spiny eel influenza virus
OD 490 nm
log10 serum dilution

Supplementary Fig. 2: No cross-reactive antibodies to bat N2 among individuals vaccinated against seasonal influenza. a Known MxA escape mutations in NP from the 1918 and the 2009 pdmH1N1 strains are highlighted in red and the resistance patch of the Eurasian avian-like swine isolate Belzig is shown in blue. Note that the avian-adapted IAV KAN-1 and bat H9N2 do not harbor any of the known MxA-resistance amino acid residues. The NP model was created with PyMol based on the available crystal structure (PDB code: 2Q06). **b** Reactivity of sera from 15 healthy adults taken before the 2022/23 seasonal influenza vaccination to recombinant N2 from bat H9N2. **c**,**d** Reactivity of the same sera against the recombinant N2 of a recent seasonal H3N2 strain (**c**) and the recombinant NA of the Wuhan spiny eel influenza virus (**d**). **e** Pre- and post- seasonal vaccination reactivity of sera from 15 healthy adults who received the 2022/23 seasonal influenza virus vaccine against recombinant N2 from the bat H9N2 virus. Reactivity was quantified as area under the curve (AUC). A paired *t*-test was used to determine statistical differences. For **b, c** and **d,** mAb 1G01 was used as positive control, an irrelevant human mAbs was used as negative control.
